# Prevalence of asymptomatic malaria and its associated factors in the Al Zuhrah district, Al-Hodeidah Government, Yemen, 2022

**DOI:** 10.1186/s12936-026-05993-y

**Published:** 2026-06-12

**Authors:** Mohammed Ahamed Hajjam, Mohammed Abdullah Al Amad, Samar Saeed Nasher

**Affiliations:** 1 Electronic Integrated Disease Early Warning System (eIDEWS), Health and Environment Office, Hodeidah, Hodeidah Governorate Yemen; 2 Department of Community Medicine, Faculty of Medicine and Health Sciences, Sana’a University, Sana’a, Yemen; 3 Electronic Integrated Disease Early Warning System (eIDEWS), Ministry of Public Health and Environment, Sana’a, Yemen

**Keywords:** Asymptomatic malaria, Prevalence, Associated factors, Al-Hodeidah, Yemen

## Abstract

**Background:**

Asymptomatic malaria represents a significant health issue, especially in endemic regions where asymptomatic individuals serve as reservoirs. In Yemen, the Al-Hodeidah governorate is the primary endemic area. This study aims to determine the prevalence of asymptomatic malaria and its associated factors in Al-Zuhra District, Al-Hodeidah Governorate.

**Methods:**

A cross-sectional community-based study was conducted using a multistage sampling method with probability proportional to size to select villages and households. In each household, one eligible member who had not experienced malaria symptoms and had consented to participate was randomly selected. Data were collected using a semi-structured questionnaire, and blood samples were tested for malaria using a Rapid Diagnostic Test. Logistic regression was used to calculate crude and adjusted prevalence odds ratios (APOR) with 95% confidence intervals (CI). A P value < 0.05 was considered statistically significant.

**Results:**

A total of 422 participants were enrolled, with a mean age of 35.7 ± 11 years; 47% were male, and 62% were from a family with more than five members. The overall prevalence of asymptomatic malaria was 5%, with *Plasmodium falciparum* accounting for 95% of infections. Being educated was protective against infection (APOR = 0.35; 95% CI 0.13–0.97; p = 0.043). Absence of window screens (APOR = 9.56; 95% CI 2.15–42.51; p = 0.003), and living near stagnant water (APOR = 4.93; 95% CI 1.50–16.23; p = 0.009) were significantly associated with asymptomatic malaria.

**Conclusion:**

Asymptomatic malaria remains present in Al-Zuhra District, with *Plasmodium falciparum* accounting for the majority of infections. Environmental and household factors, particularly absence of window screening and proximity to stagnant water were strongly associated with infection, while education showed a protective effect. These findings highlight the importance of strengthening household-level vector control measures, improving environmental management, and enhancing community awareness to reduce asymptomatic malaria transmission in the area.

**Supplementary Information:**

The online version contains supplementary material available at 10.1186/s12936-026-05993-y.

## Introduction

Malaria is still a major health concern globally and a leading cause of morbidity and mortality, particularly in low- and middle-income countries, which is caused by *Plasmodium* parasites and transmitted through the bites of infected female *Anopheles* mosquitoes [1]. Five *Plasmodium* species infect humans: *P. falciparum*, *P. vivax*, *P. ovale*, *P. knowlesi*, and *P. malariae*, of which *P. falciparum* and *P. vivax* pose the greatest public health risk [2]. Malaria infection is controllable and avoidable, but it can cause severe febrile illness and lead to life-threatening consequences if not treated properly [1, 3, 4].

Malaria presents with a spectrum of clinical manifestations that range from asymptomatic infection to life-threatening forms of the disease [5]. Asymptomatic malaria refers to he detection of the Plasmodium parasite in the blood of infected individuals who do not exhibit any clinical signs of malaria disease [5]. While symptomatic cases are more likely to be recognized and treated, asymptomatic infections are frequently overlooked and untreated, allowing infected persons to serve as silent reservoirs for ongoing malaria transmission. This buried parasite reservoir presents a poses a major obstacle to malaria elimination efforts, especially in regions where the disease is endemic [3].

Despite ongoing global control measures, malaria continues to exert a substantial burden worldwide. According to the *World Malaria Report 2025*, an estimated 282 million malaria cases and approximately 610,000 deaths occurred globally in 2024, representing an increase in cases compared with previous years and highlighting persistent challenges in malaria control [6].

In the WHO Eastern Mediterranean Region (EMRO), there were an estimated 11.1 million malaria cases and 22,100 deaths in 2024, with the burden concentrated in high-endemic countries, including Sudan, Pakistan, and Yemen [7]. *P. falciparum* and *P. vivax* remain the predominant species in the region, with *P. vivax* accounting for a large proportion of confirmed infections in EMRO settings [7].

Malaria remains endemic in Yemen; it ranks among the top ten causes of morbidity and mortality in the country, especially in coastal and lowland areas, where more than two-thirds of the population are at risk of infection [8]. According to WHO estimates, more than 21 million people in Yemen are at risk of malaria due to climatic, geographic, and socioeconomic conditions that favor transmission [9]. Since the beginning of 2024, Yemen has reported over 1,051,000 suspected malaria cases, health surveillance data documented 210,022 confirmed malaria cases with 18 related deaths [10]. In response, national efforts have included the widespread distribution of insecticide-treated nets (ITNs) and expanded diagnostic testing. Although ongoing conflict, weak health infrastructure, and limited access to services continue to challenge malaria control and elimination goals [10].

Al Hudaydah governorate is the epicenter of malaria in Yemen, accounting for approximately 66% to 80% of the country's total reported cases [11]. Despite this significant burden, reliable data on asymptomatic (silent) malaria infections in Yemen remain limited, hindering accurate estimates of the true malaria transmission reservoir in the community. Understanding the frequency of asymptomatic malaria is critical for designing surveillance and implementing effective, targeted control and elimination measures [3]. This study aims to investigate the frequency and related characteristics of asymptomatic malaria in Al Zuhra district, Al Hodeidah governorate, Yemen.

## Methods

### Study design, setting, and period

This is a cross-sectional community-based study conducted from November 21st to December 30th, 2022. Al Zuhrah district, located on the western coast of Hodeidah Governorate, Yemen. It was selected due to its hyperendemic malaria, predominantly caused by *P. falciparum* with occasional *P. vivax* infections. According to the national malaria program, this area has a high transmission rate, making it an optimal site for assessing asymptomatic malaria and its associated factors. This site was described as a tropical area, and the Morul River passes through it with two malaria transmission periods: August-April and May–September. The district has an estimated population of approximately 246,042 people and includes one hospital, three health centers, and 23 health units. Most residents engaged in agriculture, fishing, or other rural livelihoods.

### Study population

All individuals aged 18 years and older who had resided in the Al Zuhrah district for at least one year and were present during the data collection period were eligible for inclusion in the study.

### Inclusion and exclusion criteria

Participants aged 18 and older who had lived in the Al Zuhrah district for at least a year, were present during data collection, volunteered to participate in the study, and had an auxiliary body temperature of 37.5 °C at the time of data collection were eligible. Participants who were unwilling to participate, had mental illness or communication impairments, had received antimalarial therapy in the past month, or exhibited fever (≥ 38 °C) or malaria-like symptoms within the previous 48–72 h were excluded.

### Sample size

To determine the sample size, we used the single population proportion formula: (n = Z^2^ p(1-p)/ d^2^), where n is the required sample size, p is the assumed prevalence of asymptomatic malaria (50%, used as a conservative estimate in the absence of prior data to ensure maximum sample size), Z is the standard normal value with a 95% confidence interval (Z = 1.96), and d^2^ is the acceptable error (5%)[12]. The minimum sample size was 384. Following that, 10% (38) was added to account for nonresponse or missing data, resulting in a total sample size of 422 individuals.

### Sampling approach and process

A multistage sampling method was employed to choose a representative sample of the study population.

- Stage 1: A complete list of all villages in Al Zuhrah district was received from the District Health Office. Thirty villages were randomly selected.
- Stage 2: To determine the proportionate sample size for each selected village, current household demographic statistics from the local government were used. The total sample size was allocated proportionally among the selected 30 villages based on the number of households in each village.
- Stage 3: Within each selected village, households were selected using simple random sampling. From each selected household, one eligible adult per household (either the household head (male or female) or any adult member (≥ 18 years) present at the time of the visit) was randomly selected to participate in the study.

### Data collection

Data were collected through face-to-face interviews using a structured questionnaire adapted from instruments that had been validated in previous studies, with a minor alteration to meet local cultural norms [13]. The questionnaire was translated into Arabic (the local language) and validated by subject matter experts. Each interview lasted around 15–20 min.

The questionnaire was structured into three sections. The first section collected participants’ sociodemographic information. The second section focused on factors associated with asymptomatic malaria infection. The third section recorded laboratory test results used to confirm malaria infection status.

Axillary body temperature was measured by trained health professionals and community health volunteers (CHVs) to ensure that participants met the asymptomatic criteria, defined as the absence of fever at the time of data collection.

### Blood collection and analysis

Malaria infection status was detected by rapid diagnostic test (RDT) (Care Start TM Malaria HRP2/pLDH (Pf/PAN) Combo, Access Bio, New Jersey, USA), which is WHO-approved and used by the National Malaria Control Program (NMCP). Five microliters (μl) of finger-prick capillary blood were collected with a sterile lancet and deposited in the test cassette according to the manufacturer's instructions. After 20 min, the test results were examined and classified as negative, positive for *P. falciparum*, positive for *P. vivax*, or mixed. Each test cassette has a unique participant identification code. All processes followed standard operating procedures (SOPs) for biosafety and waste disposal.

### Study variables

#### Dependent variable

Asymptomatic malaria infection is characterized by the detection of malaria parasites through RDT in a person who does not have a fever (< 37.5°C) and has not experienced any malaria-related symptoms either in the two days before the survey or at the time of the assessment [13, 14].

#### Independent variable

Independent variables included sociodemographic characteristics (age, gender, education status, marital status, occupation, and family size). Educational status was categorized as uneducated (no formal schooling and unable to read and write) and educated (having attended any level of formal education). Also, factors potentially associated with asymptomatic malaria (absence or non-use of ITNs, absence of window screening, presence of stagnant water, and use of repellents). Use of repellents refers to mosquito-repelling products such as topical lotions, sprays, or coils.

#### Quality control

The questionnaire was pretested before data collection to ensure clarity and appropriateness. All diagnostic procedures and result interpretations were conducted in accordance with standard operating procedures (SOPs). RDT kits were checked for expiration dates, proper storage conditions, and physical integrity before use. Before the survey, data collectors, including three medical laboratory technicians and seven CHVs, received two days of training on interview techniques and RDT procedures. The field supervisor continuously monitored data quality through daily verification of completeness and adherence to study protocols.

### Statistical analysis

The collected data were checked, coded, and analyzed using SPSS version 28 and STATA version 14. Continuous variables were summarized using means and standard deviations, while categorical variables were presented as frequencies and percentages. Associations between asymptomatic malaria and potential factors were initially assessed using bivariate logistic regression. Variables showing statistically significant associations in the bivariate analysis (p < 0.05), along with key sociodemographic variables (age, gender, and education) as potential confounders, were included in a reduced multivariable logistic regression model to ensure model stability and prevent overfitting, following the rule of at least 10 events per variable (EPV). Crude and adjusted prevalence odds ratios (CPOR/APOR) with 95% confidence intervals were reported, and p-values < 0.05 were considered statistically significant. Findings are presented as text, tables, and graphs.

## Results

### Sociodemographic characteristics of the study respondents

Of 422 participants, almost half, 53% (222), were female, and one-third, 33% (137), were in the 35–44 age group. Nearly two-thirds 63% (264) were educated, 69% (293) were married, and 64% (268) were employed, and 62% (263) had more than five members in their household (Table 1).

**Table 1:** Sociodemographic characteristics of the study participants, 2022

Socio-demographic variables		Frequency	%
Sex	Male	200	47%
	Female	222	53%
Age	18–24	62	15%
	25–34	120	28%
	35–44	137	33%
	45–54	81	19%
	≥ 55	22	5%
	Mean (± SD)	35.7 ± 11	
Marital status	Unmarried	129	31%
	Married	293	69%
Educational status	Uneducational	158	37%
	Educational	264	63%
Occupational	Unemployed	154	36%
	Employed	268	64%
Family size	< 5	159	38%
	≥ 5	263	62%

### Prevalence of asymptomatic malaria and associated factors

**Fig. 1:**
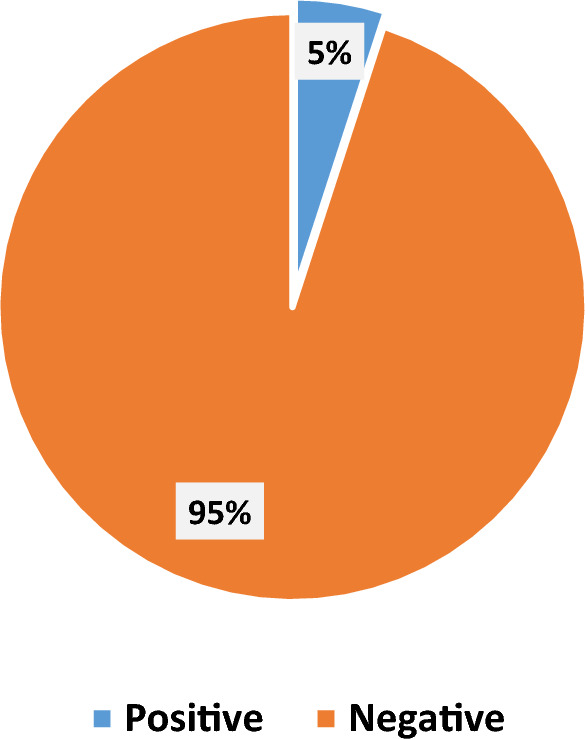
The overall prevalence of asymptomatic malaria

The prevalence of asymptomatic malaria among 422 participants was 5.0% (21/422). The *P. falciparum* accounting for 95% (20/21) of cases, and mixed infections of *P. falciparum* and *P. vivax* accounting for 5% (1/21) (Fig. 1).

### Unadjusted factors associated with asymptomatic malaria

The prevalence of asymptomatic malaria did not differ significantly between males (4%) and females (6%) (p = 0.384). Similarly, no statistically significant differences were observed across age, marital status, occupation, use of repellents, or previous history of malaria (all p > 0.05).

However, the prevalence was significantly higher among participants who did not use insecticide-treated nets (ITNs) (9%) compared to ITN users (3%). Individuals who did not use ITNs had nearly three times higher odds of asymptomatic malaria (CPOR = 2.77; 95% CI 1.14–6.70; p = 0.024). Participants living in houses without window screening had a markedly higher prevalence (25%) than those with screened windows (4%), with more than sevenfold increased odds of malaria positivity (CPOR = 7.26; 95% CI 1.81–29.13; p = 0.005). The presence of stagnant water around the household was also associated with higher prevalence (19% vs. 4%) and increased odds of asymptomatic malaria (CPOR = 5.65; 95% CI 1.89–16.93; p = 0.002) (Table 2).

**Table 2:** Prevalence of asymptomatic malaria and associated factors among study participants in Al Zuhra District, Al-Hudaydah Governorate, Yemen, 2022 Bivariate analysis

Variable		Total participant	Malaria Positive n(%)	CPOR	95% CI	P-value
Age (years)	(Continuous)	–	–	0.98	0.94–1.02	0.328
Gender	Male	200	8 (4)	(Ref)1		
	Female	222	13 (6)	1.49	0.61–3.68	0.384
Education	Not educated	158	12 (7)	(Ref)1		
	Educated	264	9 (3)	0.43	0.18–1.04	0.056
Marital status	Unmarried	129	5 (4)	(Ref)1		
	Married	293	16 (5)	1.43	0.51–4.00	0.492
Occupation	Not working	154	6 (4)	(Ref)1		
	Working	268	15 (6)	1.46	0.56–3.85	0.442
Family size	≥ 5	263	9 (3)	(Ref)1		
	< 5	159	12 (7)	2.3	0.95–5.60	0.065
Absence of ITN Home	No	268	12 (8)	(Ref)1		
	Yes	154	9 (3)	2.43	1.00–5.91	0.050
Not using ITN	No	297	10 (3)	(Ref)1		
	Yes	125	11 (9)	2.77	1.14–6.70	0.024
Absent window screening	No	410	18 (4)	(Ref)1		
	Yes	12	3 (25	7.26	1.81–29.13	0.005
Use of repellents	No	401	20 (5)	(Ref)1		
	Yes	21	1 (5)	0.95	0.12–7.46	0.963
Stagnant water around house	No	396	16 (4)	(Ref)1		
	Yes	26	5 (19)	5.65	1.89–16.93	0.002
Previous malaria	No	149	9 (6)	(Ref) 1		
	Yes	283	12 (4)	0.72	0.29–1.74	0.46

CPOR, Crude Prevalence Odds Ratio; CI, Confidence Interval; Ref, Reference category; ITN, Insecticide-Treated Net

P-values were obtained from bivariable logistic regression analysis

Statistical significance was set at p < 0.05

### Adjusted factors associated with asymptomatic malaria

To avoid overfitting, a reduced multivariable logistic regression model including only variables statistically significant in the bivariable analysis (p < 0.05), along with age, gender, and education as potential confounders, was included. The model showed good fit, as indicated by the Pearson goodness-of-fit test (χ^2^ = 161.74, p = 0.600), and explained approximately 14% of the variance in asymptomatic malaria (Pseudo R^2^ = 0.143) (Supplementary file).

In the adjusted model, living in houses without window screening was strongly associated with asymptomatic malaria, with individuals having 9.6 times higher odds compared to those with screened windows (APOR = 9.56; 95% CI 2.15–42.51; p = 0.003). The presence of stagnant water around the house was also significantly associated with higher odds of infection (APOR = 4.93; 95% CI 1.50–16.23; p = 0.009). Being educated was protective against infection (APOR = 0.35; 95% CI 0.13–0.97; p = 0.043). Not using ITNs increased the odds of asymptomatic malaria; the association was not significant (APOR = 2.53; 95% CI 1.00–6.38; p = 0.050) (Table 3).

**Table 3:** Adjusted associations of factors with asymptomatic malaria among study participants in Al Zuhra District, Yemen (Reduced multivariable logistic regression model, 2022)

Factors		APOR	(95% CI)	P value
Absent window screening	No	Ref)1		
	Yes	9.56	2.15–42.51	0.003
Stagnant water around house	No	Ref)1		
	Yes	4.93	1.50–16.23	0.009
Not using ITN	No	Ref)1		
	Yes	2.53	1.00–6.38	0.05
Education	Not educated	Ref)1		
	Educated	0.35	0.13–0.97	0.043
Age (years)	Continuous	0.96	0.92–1.01	0.143
Gender	Male	Ref)1		
	Female	1.37	0.52–3.61	0.526

APOR, Adjusted Prevalence Odds Ratio; CI, Confidence Interval; Ref, Reference category; ITN, Insecticide-Treated Net

Statistical significance was set at p < 0.05

## Discussion

Malaria is an issue of public health in many nations globally, including Yemen, which is still in the malaria control phase [8]. The results of this study indicate an overall prevalence of asymptomatic malaria of 5.0% (21/422). This result is similar to research conducted in Thailand (5%) [15] and Ethiopia (4.8%) [13]. Higher prevalence rates than our research were also found in studies performed in Yemen, notably in Bajil District (8.0%) [16], the community survey in Al Hudaydah (16.2%) [17], and Hadhramaut (13%) [18], as well as in other countries such as Nigeria (77.6%) [19] and Tanzania (8%) [20]. Conversely, lower estimates were reported in Ethiopia (3%) [21], and Haiti (1.78%) [22]. These variations may reflect differences in transmission intensity, study populations, diagnostic methods, sample size, and ecological conditions.

Regarding the causative parasite, the majority of infections (95%) were due to *Plasmodium falciparum*, which is consistent with earlier studies conducted in Yemen, particularly in the Bajil area (99%) [16]. Similar predominance of *P. falciparum* has also been reported in India (77%) [23] and Ethiopia (57.9%) [21], highlighting its dominance in most endemic settings. The presence of mixed infections (*P. falciparum* and *P. vivax*) was minimal (5%), suggesting limited circulation of non-falciparum species in the study area.

As for sex distribution, asymptomatic malaria was slightly higher among females (6%) compared to males (4%), although this difference was not statistically significant. This finding is consistent with studies from Ethiopia [23] but contrasts with reports from Ethiopia [24], Cameroon [25], and Guinea [26], where higher prevalence among males was observed. The observed variation may be explained by the higher proportion of female participants in the study, likely due to their increased availability at home during data collection, rather than true biological differences.

The present study found that environmental and household factors were the most important predictors of asymptomatic malaria. Specifically, living in houses without window screening was significantly associated with infection (p < 0.05), which aligns with findings from Tanzania [27], where the lack of protective housing structures increased malaria risk.

Similarly, the presence of stagnant water around households was significantly associated with asymptomatic malaria (p < 0.05), consistent with studies conducted in Yemen [16, 18], and Ethiopia [28], emphasizing the role of mosquito breeding sites in sustaining transmission.

In contrast, the use of insecticide-treated nets (ITNs) was not significantly associated with asymptomatic malaria in this study (APOR = 2.53; 95% CI 1.00–6.38; p = 0.050). Although this result differs from findings in Ethiopia [13], and Nigeria [29], where ITN use was significantly protective, the borderline association observed in this study may reflect inconsistent use, variation in net quality, or differences in vector biting behavior.

From a programmatic perspective, these findings highlight that asymptomatic malaria represents an important hidden reservoir of infection in Yemen, particularly because infected individuals without symptoms are unlikely to seek treatment and may continue contributing to transmission [30–32]. This is especially critical in a high-endemic and resource-limited setting such as Yemen, where interruption of transmission is challenging [30–33].

Therefore, the National Malaria Control Program should consider strengthening active case detection strategies, particularly in high-risk communities [32–34]. This may include targeted community screening, periodic household-based surveillance in areas with stagnant water and poor housing conditions, and integration of asymptomatic screening into existing primary health care services [32, 34]. Additionally, improving community awareness on consistent ITN use and environmental management (such as drainage of stagnant water and improvement of housing structures) should be prioritized to reduce transmission at the community level [31–33].

## Limitations

This is a cross-sectional study and therefore limited, as it does not allow for the determination of disease incidence or measurement of risk and cause-and-effect relationships. Rapid tests were used to diagnose malaria among the local population. The results of this study could have been more accurate if confirmed using light microscopy and advanced molecular techniques such as polymerase chain reaction (PCR), which have higher detection capabilities than rapid tests alone. The limited time available for conducting the study also contributed to this limitation.

## Conclusions and recommendations

This study demonstrates that asymptomatic malaria persists in Al-Zuhra District, with an overall prevalence of 5%, and *Plasmodium falciparum* accounts for the vast majority of infections. Although the prevalence appears moderate, the presence of asymptomatic carriers may sustain community transmission and pose challenges for malaria elimination efforts.

Environmental and household-level factors were strongly associated with infection. In particular, the absence of window screening and the presence of stagnant water around households significantly increased the odds of asymptomatic malaria. These findings underscore the critical role of vector breeding sites and inadequate structural protective measures in maintaining transmission. Conversely, education was identified as a protective factor, suggesting that awareness and knowledge may influence preventive behaviors and exposure risk.

Strengthening integrated malaria control strategies, including improved household screening, environmental management to eliminate stagnant water, and enhanced community education, may help reduce the hidden reservoir of asymptomatic infection and support malaria control efforts in endemic settings such as Al-Zuhra District.

## Supplementary Information

Additional file 1.

Additional file 2.

## Data Availability

No datasets were generated or analysed during the current study.

## Abbreviations

WHO: World Health Organization
IRS: Indoor residual spray
LLINs: Long-lasting insecticidal nets
ITN: Insecticide-treated net
SOP: Standard operating procedures
CHV: Community health volunteers
RDT: Rapid diagnostic test
COR: Crude odd ratio
AOR: Adjusted odd ratio
CI: Confidence interval
SPSS: Statistical package for social science
eIDEWS: Electronic integrated disease early warning system
