# Supplementary material for: Prevalence of asymptomatic malaria and its associated factors in the Al Zuhrah district, Al-Hodeidah Government, Yemen, 2022
Source: Malar J. 2026 Jun 12;25:301. doi: 10.1186/s12936-026-05993-y (PMC13483621; doi:10.1186/s12936-026-05993-y)
Supplement: Supplementary file 2 [file 12936_2026_5993_MOESM2_ESM.docx]

Reduced multivariable logistic regression model for factors associated with asymptomatic malaria

:
